# Genome-Wide Identification of the Double B-Box (DBB) Family in Three Cotton Species and Functional Analysis of *GhDBB22* Under Salt Stress

**DOI:** 10.3390/plants15010109

**Published:** 2025-12-30

**Authors:** Haijun Zhang, Xuerui Wu, Jiahao Yang, Mengxue He, Na Wang, Jie Liu, Jinnan Song, Liyan Yu, Wenjuan Chi, Xianliang Song

**Affiliations:** 1 Facility Horticulture Science and Technology Innovation Center, College of Jia Sixie Agriculture, Weifang University of Science and Technology, Shouguang 262700, China; 19710016232@163.com (X.W.); 18369367959@163.com (J.Y.); mx39253762@163.com (M.H.); w17852064913@163.com (N.W.); liujie655@163.com (J.L.); jinnansong93@gmail.com (J.S.); xiaoyu602602@163.com (L.Y.); 2 State Key Laboratory of Crop Biology, Agronomy College, Shandong Agricultural University, Taian 271018, China

**Keywords:** DBBs, gene family, *Gossypium*, salt tolerance

## Abstract

Salt stress causes harm to plants through multiple aspects, such as osmotic pressure, ion poisoning, nutrient imbalance, and oxidative damage. Zinc finger proteins harboring two B-box domains, known as double B-box (DBB) proteins, constitute the DBB family. While *DBB* genes have been implicated in regulating circadian rhythms and stress responses in various plant species, their functions in cotton remain largely unexplored. The present study characterized the *DBB* gene family across the genomes of *Gossypium hirsutum* L., *Gossypium raimondii* L., and *Gossypium arboreum* L., revealing a complement of 58 members. These *DBB* genes were assigned to three separate clades based on phylogenetic analysis. Members possessing close phylogenetic relationships have similar conserved protein motifs and gene structures. All DBB proteins were predicted to be nuclear-localized, consistent with their roles as transcription factors. Furthermore, the presence of multiple cis-acting elements related to light, hormone, and stress responses in the promoters implies that *GhDBBs* are integral to cotton’s environmental stress adaptation. Expression pattern analysis indicated that the expression of *GhDBB* genes was associated with the plant’s response to multiple abiotic stresses, such as salt, drought, heat (37 °C), and cold (4 °C). The reliability of the expression data was confirmed by qPCR analysis of eight selected *GhDBBs*. Under 200 mM NaCl, Arabidopsis plants overexpressing *GhDBB22* displayed longer roots and healthier true leaves than the wild-type controls. Conversely, VIGS-mediated silencing of *GhDBB22* in *G. hirsutum* led to significantly reduced salt tolerance, accompanied by exacerbated oxidative damage. Taken together, the findings from our integrated genomic and functional analyses provide a foundational understanding of the molecular mechanisms through which proteins encoded by *DBB* genes are involved in the plant’s response to salt stress.

## 1. Introduction

Due to their sessile nature, plants must persistently endure a wide spectrum of environmental stressors across all developmental stages. Osmotic stress, ionic toxicity, and oxidative damage induced by salt stress severely impair plant growth and yield [1,2]. Transcription factors act as central regulators in plant stress responses, enhancing resilience through modulating the expression of downstream functional genes [3]. Among them, zinc finger proteins with B-box motifs (BBXs) constitute an important transcription factor family involved in photoperiod regulation and stress responses [4,5,6]. For example, Ginkgo *BBX25* improves salt tolerance in poplar [6,7], At *BBX21* promotes photomorphogenesis in Arabidopsis (*Arabidopsis thaliana* L.) [8], and heterologous expression of ryegrass *BBX3* significantly confers tolerance to salt and drought stress in yeast cells [9]. In BBX family proteins, the N-terminus typically possesses one or two B-box domains, while the C-terminus sometimes contains one CCT (CONSTANS, CO-like, and TOC1) domain [10]. According to the conserved B-box and CCT domains, these members are divided into five subfamilies [11]. Notably, members containing exactly two B-box domains are defined in double B-box (DBB) proteins, which are categorized within the DBB subfamily [12].

Eight DBB members were initially identified in *Arabidopsis thaliana*, including AtDBB1a (AtBBX18), AtDBB1b (AtBBX19), AtDBB2 (AtBBX20), AtDBB3 (AtBBX22), AtDBB4 (AtBBX23), AtSTO (AtBBX24), AtSTH (AtBBX25), and AtSTH2 (AtBBX21) [13]. The majority of DBB family members participate in light signal transduction and are involved in diverse physiological processes, including seed germination [14], environmental stress response [15], and hormone signal transduction [16]. DBB2, STH2, and DBB3 promote photomorphogenesis, whereas DBB1a, DBB1b, STO, and STH function as repressors of this process and can additionally inhibit seed germination [17]. STO and STH were shown to enhance plant salt tolerance; for instance, Arabidopsis overexpressing *AtSTO* exhibited significantly longer roots under salt stress [18]. Beyond Arabidopsis, *DBB* genes have been identified in other species, displaying responsive expression under hormonal and environmental stimuli. In *Zea mays* L., 12 *DBB* genes were identified, with their expression levels changing significantly under various hormone treatments [16]. Similarly, 10 *DBB* members were identified in *Oryza sativa* L., with multiple genes exhibiting distinct expression changes in response to hormone treatments [19]. In *Triticum aestivum* L., 27 *DBB* genes were characterized, of which 11 were up-regulated and 11 down-regulated under drought stress [20]. In *Populus trichocarpa* Torr. & Gray, 12 *DBB* genes were identified, with five showing significant induction in response to drought stress [21].

Cotton is a vital global cash crop, and its fiber yield accounts for nearly 35% of the world’s natural fiber consumption [22]. Four cotton species are commercially cultivated: *Gossypium herbaceum* L. (A_1_), *Gossypium arboreum* L. (A_2_), *Gossypium hirsutum* L. (AD_1_), and *Gossypium barbadense* L. (AD_2_). Among these, *G. hirsutum* contributes to approximately 90% of global cotton production, making it the predominant cultivated species [23]. Additionally, *G. barbadense* and *G. arboreum* remain under cultivation, whereas the production of *G. herbaceum* has become highly limited. As an allotetraploid species, *G. hirsutum* possesses a genome derived from A and D subgenomes. It is generally recognized that *Gossypium raimondii* L. (D_5_) serves as the D-genome donor to modern allotetraploid cottons among diploid species [24]. *G. arboreum* (A_2_) is considered the closest extant relative of the A-subgenome donor in upland cotton, having diverged from the extinct progenitor (A_0_) approximately 700,000 years ago [25]. Due to its moderate salinity tolerance, cotton is widely cultivated to rehabilitate and utilize saline-alkaline arid lands. However, continuous salt-alkali stress significantly reduces the yield and fiber quality of cotton [26]. Consequently, identifying and functionally characterizing salt-tolerant genes is pivotal for breeding novel cotton varieties with enhanced resilience.

The DBBs have been systematically identified in multiple plants, and numerous members have been demonstrated to function in abiotic stress responses. However, the DBBs in cotton remain systematically uncharacterized. Therefore, it is reasonable to hypothesize that DBBs in cotton also play a crucial role in mediating responses to abiotic stress. In this study, we performed genome-wide identification of the DBB family in *G. hirsutum*, *G. raimondii* and *G. arboreum*. We identified 58 DBBs and characterized their physicochemical properties, subcellular localization, chromosomal locations, phylogeny, motifs, gene structures, gene collinearity, cis-acting elements, and expression patterns. To validate the transcriptomic data, qPCR was performed on a subset of *GhDBBs* under salt stress. The salt tolerance of Arabidopsis overexpressing *GhDBB22* and upland cotton with silenced *GhDBB22* was identified to further characterize the potential of DBB family genes in salt tolerance function. These findings thus lay the groundwork for future functional studies of DBB genes in cotton.

## 2. Results

### 2.1. Identification of DBB Members in Three Cotton Species

**Figure 1:**
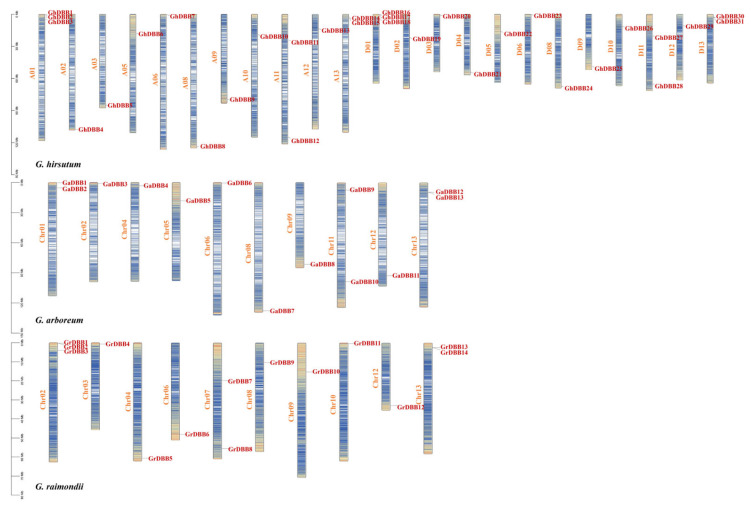
Physical location of *DBBs* on chromosomes of *Gossypium hirsutum*, *Gossypium arboreum*, and *Gossypium raimondii*. The distribution of color blocks on the chromosome represents gene density.

Proteins containing only two B-box domains were classified as DBB members, totaling 58 *DBB* genes across the three cotton species. Specifically, *Gossypium arboreum*, *Gossypium raimondii*, and *Gossypium hirsutum* harbored 13, 14, and 31 *DBBs*, respectively. The gene IDs and physicochemical properties of all *DBBs* were detailed in Table S1. All *DBB* genes were distributed across most chromosomes in the three cotton species, excluding Chr03, Chr07, and Chr10 in *G. arboreum*; Chr01, Chr05, and Chr11 in *G. raimondii*; and A03, A07, and D07 in *G. hirsutum* (Figure 1). A unified nomenclature was applied to all *DBBs*, whereby each gene was named in accordance with its linear order and precise physical location on the respective chromosome. The DBB proteins varied in length from 122 to 309 amino acids (aa). The molecular weights (MW) of GhDBBs, GaDBBs, and GrDBBs ranged from 13,391.57 to 33,698.76 Da, 13,391.57 to 33,786.87 Da, and 13,391.57 to 33,522.58 Da, respectively. The predicted isoelectric points (pI) indicated that the majority of DBBs were acidic proteins (pI 4.69–6.78), with the exception of GrDBB9 (pI = 8.37), GhDBB13 (pI = 7.6), GhDBB29 (pI = 7.14), and GaDBB11 (pI = 7.16). The aliphatic indices of all DBBs ranged from 58.1 to 90.33. The instability index of most DBBs ranged from 40.28 to 68.06, which predicted that they were unstable proteins. However, four GhDBBs (GhDBB1, GhDBB8, GhDBB16, GhDBB24), one GaDBB (GaDBB7), and one GrDBB (GrDBB5) were classified as stable proteins. According to the grand average of hydropathicity results, GhDBB8, GhDBB24, GrDBB5, and GaDBB7 were classified as hydrophobic proteins, while the remainder were hydrophilic. A nuclear localization was predicted for every identified DBB protein, which was consistent with their function as transcription factors.

### 2.2. Phylogenetic Relationship of DBBs

**Figure 2:**
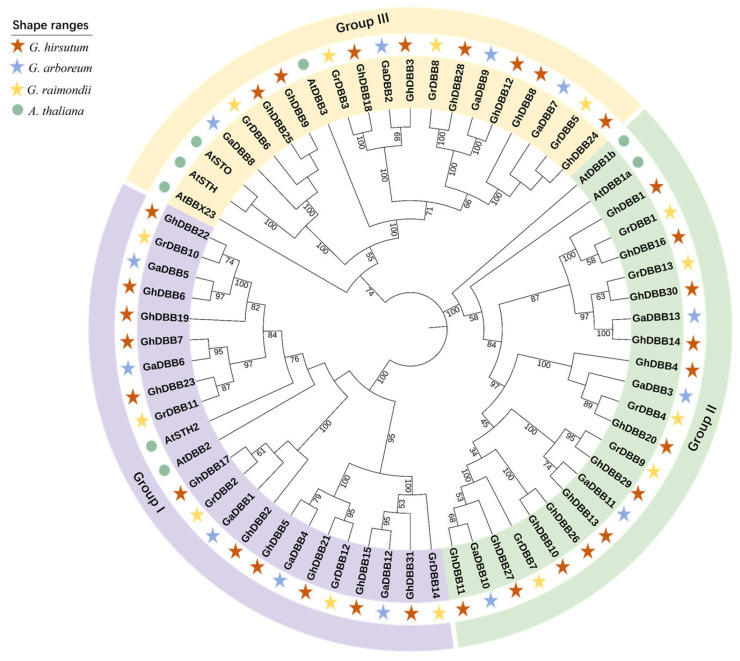
Phylogenetic trees of DBB amino acids in *A. thaliana*, *G. hirsutum*, *G. arboreum*, and *G. raimondii*. The four species are marked with graphics of different colors.

A maximum-likelihood phylogenetic tree was generated using 8 DBB proteins from Arabidopsis (*Arabidopsis thaliana*) and 58 from three cotton species to elucidate their evolutionary relationships (Figure 2). All DBBs were classified into three major groups (I–III). These three branches all contained DBBs of Arabidopsis. Groups I and II each contained 21 cotton DBBs, while Group III contained the fewest, with only 15 cotton DBBs. Phylogenetic analysis of the DBB genes provided evidence supporting the allotetraploid origin of *G. hirsutum* (AD_1_). Specifically, DBBs encoded by the A subgenome primarily clustered with orthologs from *G. arboreum* (A_2_), while those encoded by the D subgenome clustered with orthologs from *G. raimondii* (D_5_).

### 2.3. Gene Structure and Motifs in DBBs

**Figure 3:**
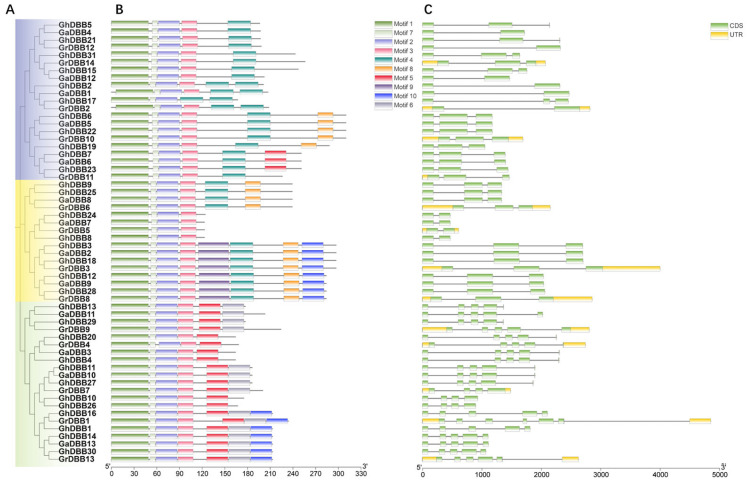
The gene structure and conserved motifs of DBBs in *G. hirsutum*, *G. arboreum*, and *G. raimondii*. (**A**) Evolutionary trees were constructed using DBB protein sequences from three cotton species. Groups I, II, and III are highlighted in purple, green, and yellow. (**B**) The motifs distribution of DBBs. (**C**) The genetic structure distribution of DBBs. The reference genome annotations for *G. arboreum* and *G. hirsutum* lacked UTR information.

To further analyze the differences among different groups, we conducted an analysis of gene structure and conserved motifs. This revealed that all members of the DBB family contained introns, and the exon number varied between 2 and 7 among the three cotton species (Figure 3). Specifically, 10 and 27 genes were found to have 2 and 3 exons, respectively. DBBs in Group I and Group III typically had 2 to 3 exons, whereas those in Group II generally contained 5 to 7 exons. The conserved group-specific patterns in gene structure provided additional evidence for the phylogenetic divergence among these clades.

Analysis of conserved motifs identified ten distinct amino acid sequences across all DBB proteins in the three cotton species using MEME. The specific sequences of motifs are shown in Table S2. As expected, Motif 1 and Motif 2, which are present in all DBBs, corresponded to the two characteristic B-box domains. Notably, Motif 7 was consistently located between Motif 1 and Motif 2, suggesting a potential structural or functional role within the core domain architecture. With the exception of GhDBB17, Motif 3 was also ubiquitous. The distribution of other motifs exhibited clear group-specific patterns. Motif 4 and Motif 5 were specific to Group I and Group II, respectively. Motif 8 was exclusively detected in Groups I and III, and Motif 6 was restricted to Group II. This orderly distribution of motifs provides additional support for the reliability of the evolutionary relationships inferred from the phylogenetic tree.

### 2.4. Gene Replication and Collinearity Analysis of DBBs in Cotton

**Figure 4:**
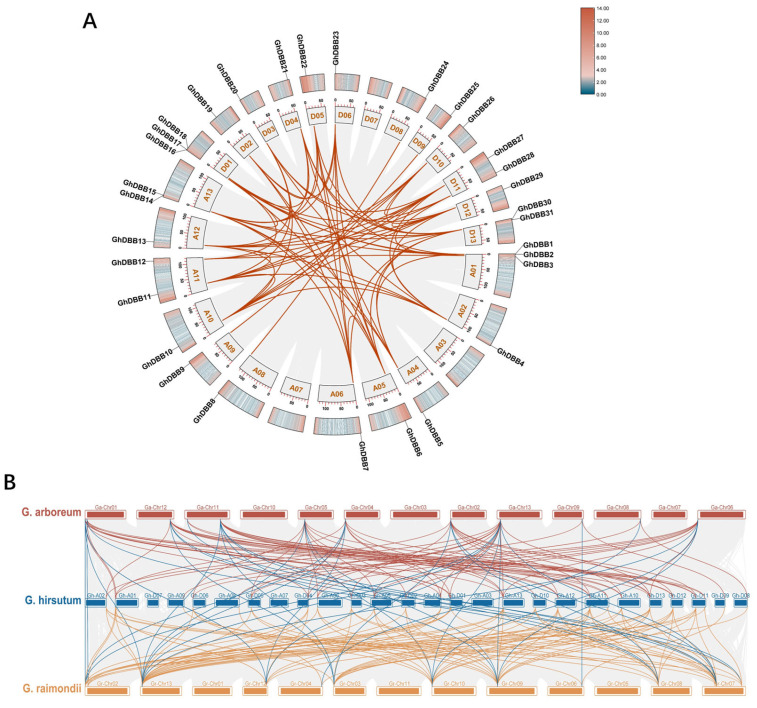
Collinearity analysis of *DB*Bs in *G. hirsutum*, *G. arboreum*, and *G. raimondii*. (**A**) Intraspecific collinearity of *GhDBBs*. Homologous *GhDBB* gene pairs are linked by red lines in the synteny plot. The outer circle shows the gene density, with red and blue corresponding to high and low gene abundance, respectively. (**B**) Interspecific collinearity of *DBB* genes among three cottons. Red lines denote collinear pairs between *GhDBBs* and *GaDBBs*; orange lines denote collinear pairs between *GhDBBs* and *GrDBBs*; blue lines denote collinear pairs between *GaDBBs* and *GrDBBs.*

Collinearity analysis of homologous gene pairs in *G. hirsutum* (AD_1_), *G. arboreum* (A_2_), and *G. raimondii* (D_5_) revealed a total of 201 collinear relationships among *DBBs* in these three species (Figure 4B). Specifically, 70 collinear pairs were detected between *GhDBBs* and *GaDBBs*, and 94 were detected between *GhDBBs* and *GrDBBs*. The larger number of collinear pairs with *G. raimondii* indicated closer evolutionary proximity to *G. hirsutum*. This supports the previous conclusion that *G. raimondii* was the D-genome ancestor of allotetraploid, while the A-genome ancestor was derived from an extinct diploid ancestor (A_0_) [25]. Furthermore, 37 collinear pairs were identified between the two diploid species, suggesting that the expansion of the *DBB* gene family commenced prior to the divergence of the A- and D-genome diploid lineages. Collinearity analysis revealed that the *DBBs* were predominantly shaped by WGD events, with no evidence of tandem duplication. This result underscores the predominant role of WGD in driving the evolution and augmentation of the *DBBs* in cotton.

To gain deeper insights into the evolutionary history of the *DBBs* in *G. hirsutum*, we analyzed intraspecific collinearity within the *G. hirsutum* genome (Figure 4). This analysis identified 70 collinear pairs of *GhDBB* genes, all of which were the result of segmental duplication. To further evaluate the selection pressures during evolution, we calculated the synonymous substitution rate (Ks) and non-synonymous substitution rate (Ka). The Ka/Ks ratios of 68 pairs of orthologous genes were all less than 1, indicating that *DBBs* underwent predominantly purifying selection during evolution, which maintains protein function conservation. This finding further confirms the dominant role of segmental duplication in the expansion of gene families.

### 2.5. The Distribution of Cis-Acting Elements in the Cotton DBB Promoters

**Figure 5:**
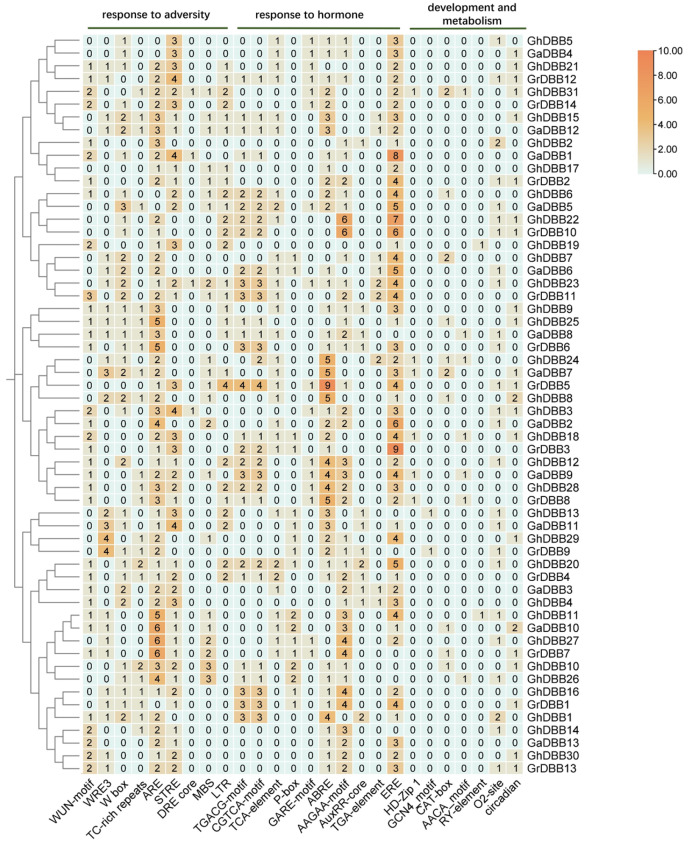
Distribution of cis-acting elements in *G. hirsutum*, *G. arboreum*, and *G. raimondii* promoters. The heatmap depicts elements associated with stress response, hormone response, and growth and development. Color intensity in each cell corresponds to the relative abundance of each element, with red denotes a higher abundance. The number in each color block indicates the specific quantity of the component.

The distribution of cis-regulatory elements within the promoter regions of three cotton species *DBB* genes was analyzed. In addition to being enriched in core promoter elements, the promoters of *DBB* genes also harbored an abundance of light-responsive elements (Table S6). Furthermore, promoters harbored elements linked to stress and hormone responses, and plant development. These elements were statistically summarized and visualized in a heatmap (Figure 5). The results demonstrated that the vast majority of *DBB* promoters contained antioxidant response elements (AREs), which are associated with cellular redox regulation. Cis-acting elements involved in defense responses, including STRE, WUN-motif, and WRE3, were abundant in *DBB* promoters. The *DBB* promoters also harbored cis-acting elements related to environmental stress, including dehydration (DRE core), low-temperature (LTR), and drought response elements (MBS). The presence of these stress and strain elements suggested that most members of this gene family could respond to adverse pressures. Among all hormone response elements, those associated with ethylene (ERE) and abscisic acid (ABRE and AAGAA-motif) were the most prevalent in *DBB* promoters. In addition, some jasmonic acid (TGACG-motif, CGTCA-motif), salicylic acid (TCA-element), and gibberellin response elements (P-box, GARE-motif) were also distributed in the *DBB* promoters. In contrast to the abundant stress and hormone elements, regulatory elements for growth and development were sparsely distributed. This pattern indicated that the *DBBs* were primarily involved in regulating responses to environmental stresses.

### 2.6. Expression Characteristics of GhDBBs

**Figure 6:**
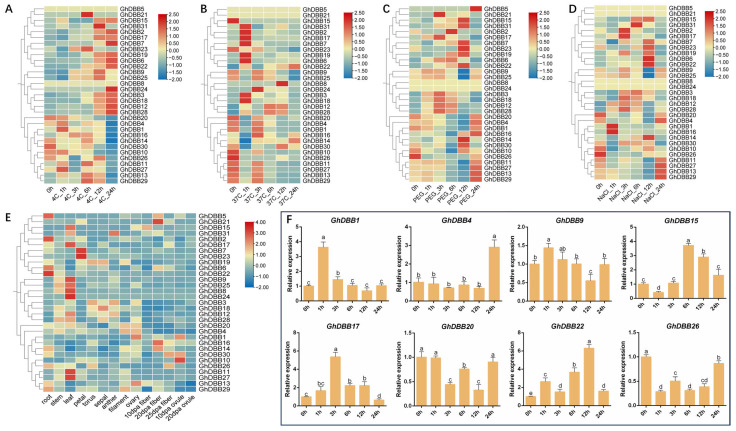
Expression profiles of *GhDBBs* in response to various stresses and across tissues. Expression patterns of *GhDBBs* under (**A**) 4 °C, (**B**) 37 °C, (**C**) 20% PEG6000 stress, and (**D**) 400 mM NaCl stress. (**E**) Transcriptional abundance of *GhDBBs* in different tissues. (**F**) Expression profiles of eight *GhDBBs* under 400 mM NaCl treatment. The sampling times were 0, 1, 3, 6, 12 and 24 h after processing. The results are the average of three repetitions. Vertical bars represent the standard deviation (±SD) from three biological replicates, and significant differences (*p* < 0.05, Student’s *t*-test) are denoted by different letters above the bars.

An analysis of *GhDBBs*’ expression across various tissues and under multiple abiotic stress conditions was performed to explore their potential biological functions. The heatmap of transcriptome data showed that most *GhDBBs* were expressed in all examined organs, albeit at varying levels (Figure 6E). *GhDBBs* preferentially expressed in leaves formed the largest group, comprising 32% of the total, followed by those in roots at 16%. Additionally, several *GhDBBs* were preferentially expressed in reproductive tissues, such as petals, ovaries, and ovules. These indicated that *GhDBBs* play roles throughout all stages of cotton growth.

The expression patterns of *GhDBBs* under abiotic stress demonstrated their widespread responsiveness. Following cold treatment, most *GhDBBs* were up-regulated, peaking at 12 or 24 h post treatment (Figure 6A). A small number of genes, such as *GhDBB14* and *GhDBB30*, exhibited down-regulated expression pattern. Following high-temperature treatment, most *GhDBBs* exhibited a transient up-regulation, followed by a progressive down-regulation (Figure 6B). A subset of genes, including *GhDBB22*, *GhDBB28*, and *GhDBB12*, remained stably up-regulated throughout the high-temperature stress treatment. Following drought stress, most *GhDBBs* peaked at 12 or 24 h, while some genes (*GhDBB3*, *GhDBB12*, *GhDBB18* and *GhDBB28*) reached their peak expression at 3 h after stress and then gradually decreased (Figure 6C). The expression patterns after salt stress treatment were more diverse (Figure 6D). *GhDBB1* and *GhDBB16* were rapidly induced within 1 h but returned to baseline levels shortly thereafter. In contrast, some genes such as *GhDBB6 and GhDBB22* slowly increased their expression levels after salt stress, reaching the highest expression at 12 h. Finally, *GhDBB4*, *GhDBB13*, *GhDBB26*, *GhDBB27*, and *GhDBB9* displayed a biphasic response, characterized by an initial down-regulation followed by subsequent up-regulation.

To validate the RNA-seq data and capture the diverse expression responses within the *GhDBB* family, eight *GhDBB* genes were selected for qPCR analysis (Figure 6F). These genes were chosen to represent a spectrum of expression patterns under salt stress, including strongly up-regulated, down-regulated, and minimally altered members, based on the preliminary transcriptome data. Among them, the expression of *GhDBB1*, *GhDBB4*, *GhDBB9*, *GhDBB17*, and *GhDBB22* was significantly up-regulated. In contrast, *GhDBB20* and *GhDBB26* showed a down-regulated expression trend. *GhDBB15* displayed a transient down-regulation at 1 h, followed by a gradual increase that peaked at 6 h. *GhDBB1* was rapidly up-regulated at 1 h after stress but returned to baseline levels shortly thereafter, indicating a transient response. In contrast, *GhDBB4* exhibited the highest expression level at 24 h after salt treatment, while there was no significant difference in other time periods compared with that at 0 h. Among the eight genes tested, *GhDBB22* showed the greatest fold change under salt treatment, with a 6.3-fold increase over the 0 h control at 12 h. Collectively, these qRT-PCR results demonstrate that most members of the *GhDBBs* are involved in the plant’s response to salt stress.

### 2.7. Heterologous Expression of GhDBB22 Enhanced Salt Tolerance in Arabidopsis

**Figure 7:**
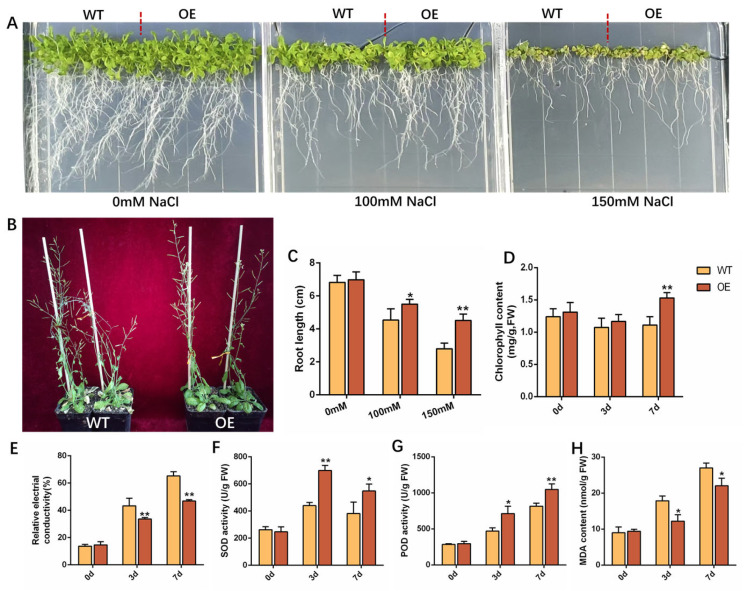
Heterologous expression of *GhDBB22* confers enhanced salt stress tolerance in Arabidopsis. (**A**) Phenotypes of WT and OE plants grown for 20 days under 0, 100, and 150 mM NaCl. (**B**) Phenotypes of Arabidopsis after 7 days of 200 mM NaCl stress. (**C**) Root length of Arabidopsis under stress for 20 days. (**D**) Chlorophyll content, (**E**) relative electrical conductivity, (**F**) SOD activity, (**G**) POD activity, and (**H**) MDA content in Arabidopsis leaves after 7 days of 200 mM NaCl treatment. Significant differences are indicated by * (*p* < 0.05) and ** (*p* < 0.01).

Given that *GhDBB22* showed the highest fold upregulation under salt stress, we generated Arabidopsis overexpression lines (OE) in the wild-type (WT) to functionally characterize its role in salt tolerance. Both WT and OE lines were grown on 1/2 MS medium and treated with 0, 100, or 150 mM NaCl for 20 days to induce salt stress. Under 0 mM NaCl, the growth phenotypes of OE and WT lines was comparable (Figure 7A). Under salt stress (100 and 150 mM NaCl), however, the OE line exhibited significantly longer roots than WT plants. This effect was most pronounced at 150 mM NaCl, where the root length of OE plants was approximately twice that of WT plants (Figure 7C).

One-month-old Arabidopsis was used to further study the salt tolerance of OE Arabidopsis at the adult plant stage. After irrigation with 200 mM NaCl for 7 days, WT plants exhibited severe leaf chlorosis and necrosis, accompanied by the wilting of flower stalks (Figure 7B). In contrast, OE lines exhibited only minor wilting in a small number of rosette leaves, while most leaves and flower stalks remained healthy. At this time, the chlorophyll content of OE lines was 1.53 mg/g, while that in WT lines was only 1.11 mg/g (Figure 7D). The relative electrical conductivity (REC) of WT plants (65.26%) was higher than that of OE plants (46.84%) (Figure 7E). In addition, after salt stress, the superoxide dismutase (SOD) activity (Figure 7F) and peroxidase (POD) activity (Figure 7G) of OE plants were significantly higher than those of WT, and the malondialdehyde (MDA) content (Figure 7H) was significantly lower than that of WT plants. These results indicated that OE plants exhibited enhanced salt tolerance.

### 2.8. Silencing of GhDBB22 Impaired Salt Tolerance in Upland Cotton

**Figure 8:**
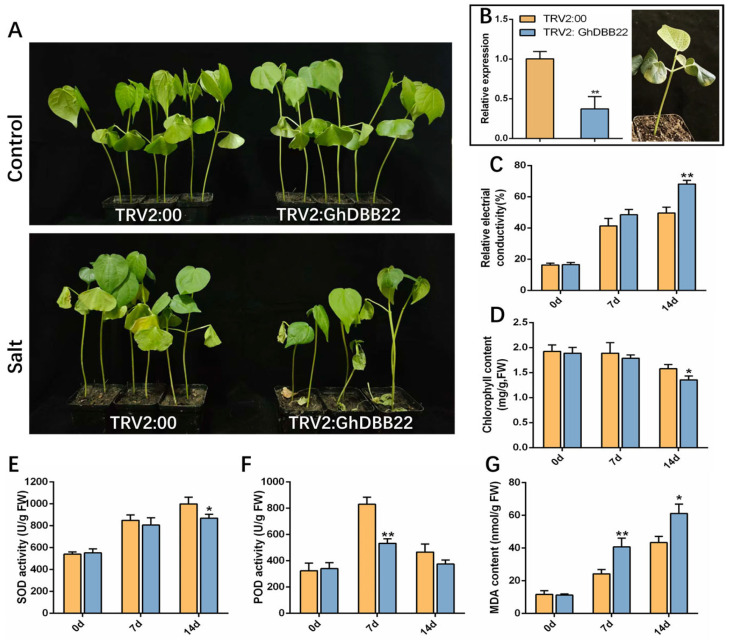
Silencing of *GhDBB22* compromises salt tolerance in cotton. (**A**) Cotton plant phenotypes after 14 days of 300 mmol/L NaCl immersion. (**B**) *GhDBB22* gene silencing efficiency and the albino phenotype of the plants infected with TRV2:*GhCLA1*. Relative electrical conductivity (**C**), total chlorophyll content (**D**), SOD activity (**E**), POD activity (**F**) and MDA content (**G**) of leaves were changed after salt treatment. Data are presented as the mean ± SD of three biological replicates. * (*p* < 0.05) and ** (*p* < 0.01) showed significant differences.

In this study, VIGS was employed to silence *GhDBB22* in upland cotton to investigate its contribution to salt tolerance. Two weeks after Agrobacterium-mediated infection of cotyledons, the transcript abundance of *GhDBB22* in TRV2:*GhDBB22* plants decreased by 62.7%, confirming efficient silencing (Figure 8B). Plants were subsequently subjected to salt stress through irrigation with 300 mM NaCl solution, while controls were treated with water. Under well-watered conditions, no significant growth differences were observed between TRV2:00 and TRV2:*GhDBB22* plants. After 14 days of 300 mM NaCl stress, the leaves of TRV2:00 plants remained relatively fresh and turgid, and their cotyledons had not fully abscised. In contrast, the leaves of TRV2:*GhDBB22* plants exhibited severe wilting, and their cotyledons had withered and abscised (Figure 8A). After 14 days of salt treatment, the relative electrical conductivity (Figure 8C) and MDA content (Figure 8G) in the leaves of TRV2:*GhDBB22* cotton were significantly higher than those in the empty vector control plants, while the chlorophyll (Figure 8D) content was significantly lower than that in TRV2:00 plants. These results indicated that *GhDBB22* silencing exacerbated salt-induced cellular damage and impaired photosynthetic efficiency. The antioxidant capacity was compromised in TRV2:*GhDBB22* plants, as evidenced by significantly lower POD activity (Figure 8F) after 3 days and reduced SOD activity (Figure 8E) after 14 days of salt stress compared to controls. Collectively, these findings establish that *GhDBB22* is essential for protecting cotton leaves from salt-induced cellular damage and maintaining photosynthetic efficiency.

## 3. Discussion

The DBB proteins, a subfamily of B-box family, are well established as crucial regulators of circadian rhythms and early photomorphogenesis throughout the plant kingdom [27,28]. In addition to these functions, DBB family genes also mediate plant stress responses and hormone signaling pathways in diverse plant species [20,21]. To date, 8, 27, 12, and 12 DBBs have been identified in *Arabidopsis thaliana* [10], *Triticum aestivum* [20], *Populus trichocarpa* [21], and *Zea mays* [16], respectively. However, the *DBB* gene family in cotton remains uncharacterized at the genome-wide level. In this work, we identified 13, 14, and 31 DBBs in *G. arboreum*, *G. raimondii*, and *G. hirsutum*, respectively. The 58 DBBs in cotton were classified into three groups (I–III) based on their phylogenetic relationships. Genes within the same group exhibited remarkably high similarity in both gene structure and motif composition, suggesting that they may have developed relatively conserved protein functions during the evolutionary process [29]. As zinc finger transcription factors, most cotton DBBs were acidic, heat-labile proteins, and all were predicted to localize in the nucleus, consistent with their putative roles as transcription factors [30,31]. Notably, in *Solanum tuberosum* L., StDBB is localized in the nucleus at the beginning of the photoperiod but shifts to the cytoplasm at the end of the photoperiod [27]. This dynamic pattern suggests that the function of DBB proteins may be regulated by environmental conditions, raising the question of whether cotton DBBs exhibit similar changes in subcellular localization that warrant further investigation.

In the allotetraploid species *G. hirsutum*, the number of DBBs was 31, which was close to the total number of DBBs in the two diploid species *G. arboreum* (13) and *G. raimondii* (14). This finding supports the established origin of allotetraploid cotton, which arose from the hybridization and genome doubling of A- and D-genome diploid species [25]. Gene collinearity is defined as a conserved linear order of homologous genes across chromosomes within or between genomes, and it helps reveal phylogenetic relationships [32]. Our analysis revealed a greater number of collinear DBB pairs between *G. hirsutum* and *G. raimondii* (94) than between *G. hirsutum* and *G. arboreum* (70), indicating a closer genetic relationship with *G. raimondii*. This result aligns with the conclusion that *G. raimondii* is the direct progenitor of the tetraploid D-genome, while *G. arboreum* is a collateral relative of the A-genome ancestor [25]. Regarding gene family expansion, we found no tandemly duplicated genes in the cotton DBB family, suggesting that WGD served as the primary driving force [33,34]. After each gene duplication event, these genes are either retained or eliminated during evolution [35]. The Ka/Ks ratios for 97% of the DBB orthologous gene pairs in *G. hirsutum* were less than 1, indicating that this gene family has undergone strong purifying selection with retention of primarily beneficial variations [36].

Phytohormones play a crucial role in regulating plant physiological activities and responding to stressful environments, serving as core signaling molecules in growth and metabolic processes [37,38]. *DBBs* are believed to be involved in hormone responses in various plants. This is evidenced by studies showing that the expression of 12 *PtrDBBs* was altered under ABA and MeJA stress in poplar and that of 27 *TaDBBs* was differentially expressed under ABA stress in wheat [20,21]. A diversity of hormone-responsive cis-elements was identified in the *GhDBB* promoters, including those related to ethylene, jasmonic acid, salicylic acid, and gibberellin signaling. This suggests that cotton DBBs may also be involved in multiple hormone response pathways. In Arabidopsis, *AtSTO* (*AtBBX24*) confers improved salt and drought tolerance via the ABA response pathway [18,39]. Mutants of Arabidopsis *sth2* (*bbx21*) exhibited hypersensitivity to ABA and salt stress, which implicated this gene in the corresponding stress response pathways [40]. Except for *GhDBB19* and *GhDBB21*, the promoters of the remaining cotton *DBBs* contained abscisic acid-responsive elements (ABRE and AAGAA-motif), suggesting that the majority of cotton *DBB* family genes may play important roles in abscisic acid response pathways.

Furthermore, the promoters of *GhDBBs* were enriched with numerous stress-responsive cis-acting elements, including dehydration response elements (DRE core), drought response elements (MBS), and low-temperature response elements (LTR). This indicates the important function of DBBs in plants’ response to adverse stress. *AtDBB1a* (*AtBBX18*) and *AtDBB4* (*AtBBX23*) promoted heat-responsive growth by increasing the accumulation of their transcripts and proteins under high-temperature conditions [41,42]. *AtSTH* (*AtBBX25*) and *AtSTO* (*AtBBX24*) have been shown to confer enhanced salt tolerance in plants [18], while heterologous overexpression of *AtSTH2* (*AtBBX21*) has been demonstrated to enhance drought tolerance in potato [43].

In this study, most *GhDBBs* were up-regulated under various abiotic stresses, including low temperature, high temperature, drought, and salt, suggesting their positive roles in stress adaptation. This is similar to the findings in poplar, wheat and corn [16,20,21]. The promoter of *GhDBB22* from Group I contained multiple hormone and stress-responsive elements, and this gene was upregulated in transcriptome analyses under four different abiotic stresses. qPCR analysis confirmed that the expression of *GhDBB22* was induced by salt stress. To directly assess its role in salt tolerance, we generated *GhDBB22*-overexpressing lines in Arabidopsis and silenced its expression in cotton via VIGS. Under salt stress, overproduction of reactive oxygen species (ROS) induces oxidative damage, which is typically mitigated by antioxidant enzymes including SOD and POD [44]. The extent of cellular injury can be assessed by MDA content and relative electrical conductivity (REC) [45]. Under salt stress, *GhDBB22*-overexpressing Arabidopsis lines showed improved growth, elevated antioxidant enzyme activities, and reduced oxidative damage than wild-type plants. In previous study, overexpression of *Chimonanthus praecox* (L.) Link *CpBBX19* in Arabidopsis, coupled with assessment of comparable physiological indicators, enhanced salt tolerance in the transgenic plants [46]. In contrast, *GhDBB22*-silenced cotton plants exhibited compromised plant growth and antioxidant enzyme activity, along with elevated cellular damage, compared to the control plants. Collectively, these results establish a role for *GhDBB22* in enhancing plant salt tolerance. Future work should aim to elucidate the precise molecular pathways through which *DBB* genes mediate this stress response.

## 4. Materials and Methods

### 4.1. Identification of DBB Family Members

The genomic resources utilized in this study were obtained from two public cotton-specific databases. The annotation and genome files of *G. hirsutum* (ZJU) [34], *G. raimondii* (JGI) [35], and *G. arboreum* (CRI) [47] were obtained from COTTONOMICS. The sequencing version number is presented in parentheses. Based on existing research and genome annotation information, eight DBB proteins of *A. thaliana* were acquired from TAIR [13]. All the website addresses of the online tools used are recorded in Table S11.

DBBs in cotton were identified through a combined BLAST and HMMER search using TBtools-II (v2.376) (Hereinafter referred to as Tbtools) [48,49]. First, we performed a BLASTP search against the proteomes of three cotton species using eight Arabidopsis DBB protein sequences as queries (E-value cutoff: 1 × 10^−5^). Concurrently, the Hidden Markov Model profile for the B-box domain (PF00643) was obtained from Pfam website database and used to search the same proteomes via HMMER implemented in TBtools. The candidate sequences from both methods were merged, and redundant entries were removed. All the obtained sequences were verified using the NCBI Conserved Domain Database, and the sequences that did not conform to the characteristics of the DBB family were deleted. Finally, proteins containing exactly two B-box domains were retained as the definitive DBB family members for subsequent analysis.

### 4.2. Analysis of the Physicochemical Properties and Chromosomal Physical Location Distribution of DBB Family Members

The Protein Parameter Calc tool built into TBtools was used to predict the physicochemical properties of amino acids for all DBBs. Subcellular localization prediction of all DBBs was carried out using the Plant-mPLoc website. The chromosomal locations of DBBs were visualized with the Gene Location Visualize From GTF/GFF in TBtools [48]. Finally, systematic naming was carried out based on the arrangement order of DBBs on chromosomes.

### 4.3. Phylogenetic Analysis of DBBs

First, sequence alignment of the DBBs was performed using the MUSCLE Wrapper plugin in TBtools. The resulting alignment was then trimmed using the trimAL Wrapper plugin. A maximum-likelihood phylogenetic tree was constructed with the IQ-tree Wrapper, which employed the auto model and 1000 bootstrap replicates. Finally, beautify the evolutionary tree using the interactive Tree of Life website.

### 4.4. Analysis of the Gene Structure Characteristics and Motifs of DBBs

Based on the gene annotation file and *DBBs* gene IDs, the gene structure was visualized using the Gene Structure View function of TBtools. Identify the motifs of DBBs using the Multiple Em for Motif Elicitation online platform. According to the method described earlier, the phylogenetic trees of DBBs in three cotton species were constructed. Finally, the phylogenetic tree, gene structures, and conserved motifs were jointly imported into the Advanced Gene Structure View function of TBtools for integration, generating a comprehensive visualization map.

### 4.5. Collinearity Analysis of the DBBs in Cotton

Collinearity relationships and gene duplication events were analyzed for all pairwise combinations of cotton species using the One Step MCScanX tool in TBtools (E-value ≤ 1 × 10^−10^). The Dual Systeny Plot function was employed for visualizing interspecies collinearity, while intraspecific collinearity was visualized using Advanced Circos. To assess selection pressure, the Ka and Ks for *DBBs* in *G. hirsutum* were calculated with the Simple Ka/Ks Calculator (NG).

### 4.6. Prediction of Cis-Acting Elements of the DBBs

First, using the Gtf/Gff3 sequence extraction function of TBtools software, the sequences of 2000 bp upstream of the *DBB* start codon were obtained. Subsequently, PlantCare was used to predict the cis-acting elements contained in these sequences. After statistical analysis of the prediction results, TBtools was finally used to visualize the various elements.

### 4.7. Expression Characteristic Analysis and qPCR Verification of GhDBBs

To explore the response characteristics and tissue expression patterns of the *GhDBBs* under abiotic stress, we obtained relevant expression data from the gene expression database of TM-1 [34]. All the transcriptome expression data of *GhDBBs* were generated into heat maps using the heatmap function of TBtools, and the FPKM were standardized using Z-score normalization.

To validate the expression profile data, salt stress treatment was applied to TM-1 cotton. Cotton plants were cultured under conditions of 16 h light/8 h dark (25 °C). Four-week-old seedlings were subjected to salt stress by irrigation with 400 mM NaCl solution. Root tissues were collected after treatment. Each replicate was taken from 2 cotton plants. Plant RNA was extracted using the OminiPlant RNA Kit (DNase I) (CWBIO, Jiangsu, China) and then reverse-transcribed into cDNA. Taking *GhUBQ14* as the internal reference gene, the expression pattern of *GhDBBs* was analyzed by qPCR [50]. The primer sequences were shown in Supplementary Table S9.

### 4.8. Obtainment of Arabidopsis with Heterologous Expression of GhDBB22 and Identification of Its Salt Tolerance

The CDS of *GhDBB22* was cloned from TM-1 cDNA and linked into the PRI101-AN vector to get a 35S::*GhDBB22* plant overexpression construct. This construct was then introduced into Col-0 via the floral dip method [51]. Select the OE strain with the highest expression level of *GhDBB22*. Transgenic lines were selected on 1/2 MS medium containing 35 mg/L kanamycin, and homozygous T_3_ seeds were obtained for subsequent experiments. To assess salt tolerance at the seedling stage, seeds of WT and *GhDBB22*-overexpressing lines were sown on 1/2 MS culture medium containing 0, 100, or 150 mM NaCl. For adult plants, 30-day-old soil-grown WT and OE lines were subjected to 200 mM NaCl for 7 days. Leaf samples were collected at 0, 3, and 7 days after treatment to quantify total chlorophyll content, REC, MDA content, and the activities of POD and SOD. We measured total chlorophyll content spectrophotometrically, relative electrical conductivity with a conductivity meter, SOD activity via nitrogen blue tetrazolium photoreduction, POD activity using guaiacol colorimetry, and MDA content with the thiobarbituric acid method. All assays were performed according to established methods [52]. Two plants per pot were considered as one biological replicate, and each data point represented the mean of three independent replicates.

### 4.9. Salt Tolerance Evaluation of Upland Cotton Following GhDBB22 Silencing via VIGS

Given the salt sensitivity of the standard upland cotton line TM-1, the salt-tolerant commercial cultivar Lumianyan 37 was chosen to silence *GhDBB22* via VIGS and assess its function in salt tolerance. The non-conserved domain fragment of *GhDBB22* was cloned into the VIGS expression vector TRV2, generating the silencing construct TRV2:*GhDBB22*. The recombinant plasmid TRV2:*GhDBB22*, the empty vector TRV2:00, and the positive control TRV2:*GhCLA1* (which induces an albino phenotype to confirm successful infection) were individually introduced into *Agrobacterium tumefaciens* GV3101. At the cotyledon expansion stage of upland cotton variety Lumianyan 37, TRV1 was mixed with TRV2:00, TRV2:*GhCLA1*, and TRV2:*GhDBB22* Agrobacterium solutions each at a 1:1 volume ratio. Injected the mixed bacterial solution into the cotyledons of cotton. After infiltration, plants were kept in darkness for 24 h. When the positive control plants exhibited albino leaf phenotypes, leaves from plants injected with TRV2:00 and TRV2:*GhDBB22* solutions were collected to assess *GhDBB22* silencing efficiency via qRT-PCR.

Salt stress was applied to the plants using 300 mM NaCl solution, while control plants were watered similarly without NaCl. Leaf samples were collected at 0, 7, and 14 days after treatment initiation to measure total chlorophyll content, REC, MDA content, and the activities of POD and SOD. For each data point, two cotton seedlings per pot were considered as one biological replicate, and the mean value from three independent biological replicates was calculated.

## 5. Conclusions

According to their phylogenetic relationships and motif distributions, the 58 *DBB* family members identified in three cotton species were categorized into three groups. Gene collinearity and gene duplication events supported the origin theory of tetraploid cotton. The distribution of promoter cis-acting elements and stress response expression patterns highlighted the functions of the *DBB* gene family in cotton adaptation to abiotic stress and hormone responses. Transgenic and VIGS methods verified the function of *GhDBB22* in enhancing plant salt tolerance. These findings provide references for the potential functions of the *DBB* genes in cotton. Furthermore, this work lays a foundation for deciphering the complex regulatory network underlying cotton’s abiotic stress tolerance. The identified salt-responsive *GhDBB* members, especially *GhDBB22*, serve as pivotal starting points for future investigations into their downstream targets and crosstalk with hormone signaling pathways, and their coding sequences hold potential for genetic engineering to develop new cultivars with enhanced salt tolerance.

## Data Availability

The data can be obtained in the articles and Supplementary Materials.

## Supplementary Materials

The following supporting information can be downloaded at: https://www.mdpi.com/article/10.3390/plants15010109/s1, Table S1: The sequence properties of DBB genes identified in three cotton species; Table S2: Information on motifs in DBBs; Table S3: Interspecific collinearity of *DBBs* in cotton; Table S4: Syntenic blocks of the *GhDBB* genes in *G. hirsutum*; Table S5: Ks, Ka and Ka/Ks calculation of the duplicated *GhDBB* gene pairs; Table S6: Cis-acting element in the promoter of the *DBBs*; Table S7: The expression profiles (FPKM) of *DBBs* in *G. hirsutum*; Table S8: qRT-PCR results of *GhDBBs* under salt stress; Table S9: Specific primers for qRT-PCR or vector constructions; Table S10: The qPCR results of *GhDBB22* in transgenic Arabidopsis; Table S11: The website address used in the method; Table S12: The sequence information used in the construction of phylogenetic trees.
